# A systematic review of the safety of tirzepatide-a new dual GLP1 and GIP agonist - is its safety profile acceptable?

**DOI:** 10.3389/fendo.2023.1121387

**Published:** 2023-03-27

**Authors:** Zhuqing Meng, Min Yang, Haibo Wen, Su Zhou, Chuan Xiong, Yu Wang

**Affiliations:** ^1^ Department of Pharmacy, Mianyang Fulin Hospital, Mianyang, Sichuan, China; ^2^ Department of Pharmacy, Mianyang Central Hospital, School of Medicine, University of Electronic Science and Technology of China, Mianyang, Sichuan, China; ^3^ Department of Pharmacy, Sichuan GEM Flower Hospital, Chengdu, Sichuan, China

**Keywords:** dual glucose-dependent insulinotropic peptide and glucagon-like peptide-1 receptor agonist, tirzepatide, safety, discontinuation, dose-dependence

## Abstract

**Aims:**

Tirzepatide is a novel dual glucose-dependent insulinotropic peptide (GIP) and glucagon-like peptide-1 receptor agonist (GLP-1 RA). At present, there is no controversy over its effectiveness, but its safety. We conducted a systematic review to assess the safety of tirzepatide.

**Methods:**

We searched PubMed, Embase and Cochrane databases for randomized controlled trials (RCTs) of tirzepatide from databases inception to August 28, 2022 and used the Cochrane Systematic Assessment Manual Risk of Bias Assessment Tool (version 5.1) and modified Jadad scale to assess risk of bias. The systematic review was conducted *via* Revman5.4.

**Results:**

Nine RCTs with a total of 9818 patients were included. The overall safety profile of tirzepatide is similar to GLP-1RAs, except for the hypoglycemia (tirzepatide 15mg, pooled RR=3.83, 95% CI [1.19- 12.30], *P=0.02*) and discontinuation (tirzepatide 10mg, pooled RR=1.75,95%CI[1.16-2.63], *P=0.007* and 15mg, pooled RR=2.03, 95%CI [1.37-3.01], *P=0.0004*). It also showed that the dose escalation could not rise the occurrence rates of total, severe, gastrointestinal adverse events and hypoglycemia (*P>0.05*); Compared with 5mg, tirzepatide 10mg and 15mg were associated with more frequent nausea (*P<0.001*), discontinuation (*P<0.05*) and injection-site reaction (*P<0.01*); The rates of vomiting and diarrhea were dose-dependence at the range of 5-15mg.

**Conclusion:**

The safety profile of tirzepatide is generally acceptable, similar to GLP-1 RAs. It is necessary to pay attention to its specific adverse events (hypoglycemia and discontinuation) at high doses (10mg or higher). Nausea, vomiting, diarrhea, discontinuation and injection-site reaction were dose-dependence among specific dose ranges.As the heterogeneity in different studies by interventions, the results may be with biases and the further confirmation is needed. Meanwhile, more well-designed trials are needed to control the confounding factors and ensure adequate sample size.

## 1 Introduction

The prevalence of Type 2 diabetes mellitus (T2DM) has reached epidemic proportions and is estimated to afflict over 400 million people worldwide. Moreover, the incidence of diabetes is expected to continue to rise and, in the U.S. alone, is projected to affect nearly one in three people by the year 2050 (1). Its main harm comes from chronic irreversible damages to target organs, including cardiovascular (2), kidney (3), eyes (4), skin and soft tissues (5), etc. However, there is no cure for diabetes so far, but it can be treated and controlled by pharmacological therapy which can delay or possibly to prevent the development of diabetes-related health problems (6). It is suggested that there is a need for the development of a novel and effective treatment agent to combat the rise in T2DM prevalence worldwide.

Tirzepatide (TZP), a novel dual glucagon-like peptide-1 (GLP-1) and glucose-dependent insulinotropic polypeptide (GIP) agonist has been approved for the treatment of T2DM in United States on May 13, 2022 (7). TZP targets not only GLP-1 but also GIP receptors and/or glucagon which is intended to address different metabolic pathways for carbohydrate, lipid, and protein metabolism simultaneously (8). In terms of efficacy, almost all randomized controlled trials (RCTs) studies have shown that TZP has outstanding effectiveness in glycaemic control and weight reduction which is significantly better than GLP-1 receptor agonists (GLP-1 RAs). These results are consistent with systematic reviews (9, 10), suggesting that the efficacy of TZP is stable and unparalleled in the treatment of T2DM and obesity. In addition, the molecule including a C20 fatty acid moiety with a half-life of approximately 5 days, allowing for once-weekly subcutaneous injection which can improve the compliance of patients. All these advantages make TZP to be a milestone of anti-diabetic agents. However, the conclusions about safety remain controversial. TZP is currently considered to be as safe as GLP-1 RAs (8–10). But there are still conflicting opinions (11, 12). Meanwhile, there is no special study on safety, which may lead to inaccurate result as the small sample sizes and insufficient outcomes. As the current studies mainly focus on effectiveness, researchers seldom concern about the source of safety heterogeneity, making it difficult to evaluate TZP on a comprehensive basis.

In this manuscript, we provided a systematic review with additional studies and outcomes, and a more detailed analysis was processed. We wonder that, from the current RCTs results, whether TZP has a higher odds of adverse drug event (ADE), than placebo, insulin and especially than GLP-1 RAs; whether there is dose-dependence correlation between ADE of TZP.

## 2 Materials and methods

### 2.1 Literature search and data extraction

We performed a systematic search of PubMed, Cochrane Library, and EMBASE databases from the time of databases inception to August 28, 2022. “tirzepatide” and “safety” were used as the Medical Subject Headings (Mesh) and”LY3298176”and”safeties” as the free terms. During the retrieval, Mesh and free terms were combined for the literature search. The literature screening and data extraction were performed independently by two investigators. If there was any argument, it was resolved by a third investigator. Then, we extracted the data including the number of patients in the treatment and control groups, demographics, diseases, intervention methods, concomitant medications and treatment duration.

The outcomes are the odds of total adverse drug event (TADE), serious adverse drug event (SADE), gastrointestinal adverse drug event (GADE), discontinuation by adverse drug event (DADE), hypoglycemia and injection site reaction, etc.

### 2.2 Literature inclusion and exclusion criteria

The inclusion criteria (1) Randomized controlled trials in any published years and languages; (2) The patients in the treatment groups were given TZP at a maintenance dose of 5, 10 or 15 mg once weekly, and the patients were treated with placebo or other anti-diabetic drugs in the control groups; (3) The main outcomes meet the demand of the research.

The exclusion criteria (1) non-randomized controlled trials; (2) Animal or pharmacokinetic researches, basic studies, systematic reviews, meta-analyses, retrospective studies, case reports, or conference presentations; (3) Abstract-only publications or unpublished studies; (4) Publications missing important information;(5) Duplicate publications.

### 2.3 Assessment of risk of bias

The quality of the research was assessed according to the Cochrane Systematic Assessment Manual version 5.1 Risk of Bias Assessment Tool and the modified Jadad scale. A study with a modified Jadad scale of more than 3 was considered to be of high-quality and acceptable. The funnel plots were adopted to evaluate the risk of publication bias.

### 2.4 Statistics analysis

We calculated responder proportions with 95% confidence interval (95% CI) using the suitable model (fixed effects Mantel-Haenszel model or random effects Mantel-Haenszel model) with a double arcsine transformation. The data analysis was performed *via* RevMan 5.4 software. The pooled risk ratio (pooled RR) for safety and 95% CI for the count data measure were calculated. The heterogeneity was measured by Q test and *I* ^2^, *P >*0.10 (Q test) and *I* ^2^< 50% among all subgroups suggesting low heterogeneity and fixed effects inverse variance weight model was adopted in the statistical process; *P* ≤ 0.10 and *I* ^2^ ≥50%, heterogeneity was large and random effects inverse variance weight model was adopted; whether there was a statistically significant difference between the control and treatment groups depended on the test level (*P=0.05*). When exploring the correlation between ADE and drug dose, we used the chi-square test to evaluate whether the difference between the does groups was statistically significant, the test level was also *P=0.05*.

## 3 Results

### 3.1 Study characteristics

**Figure 1:**
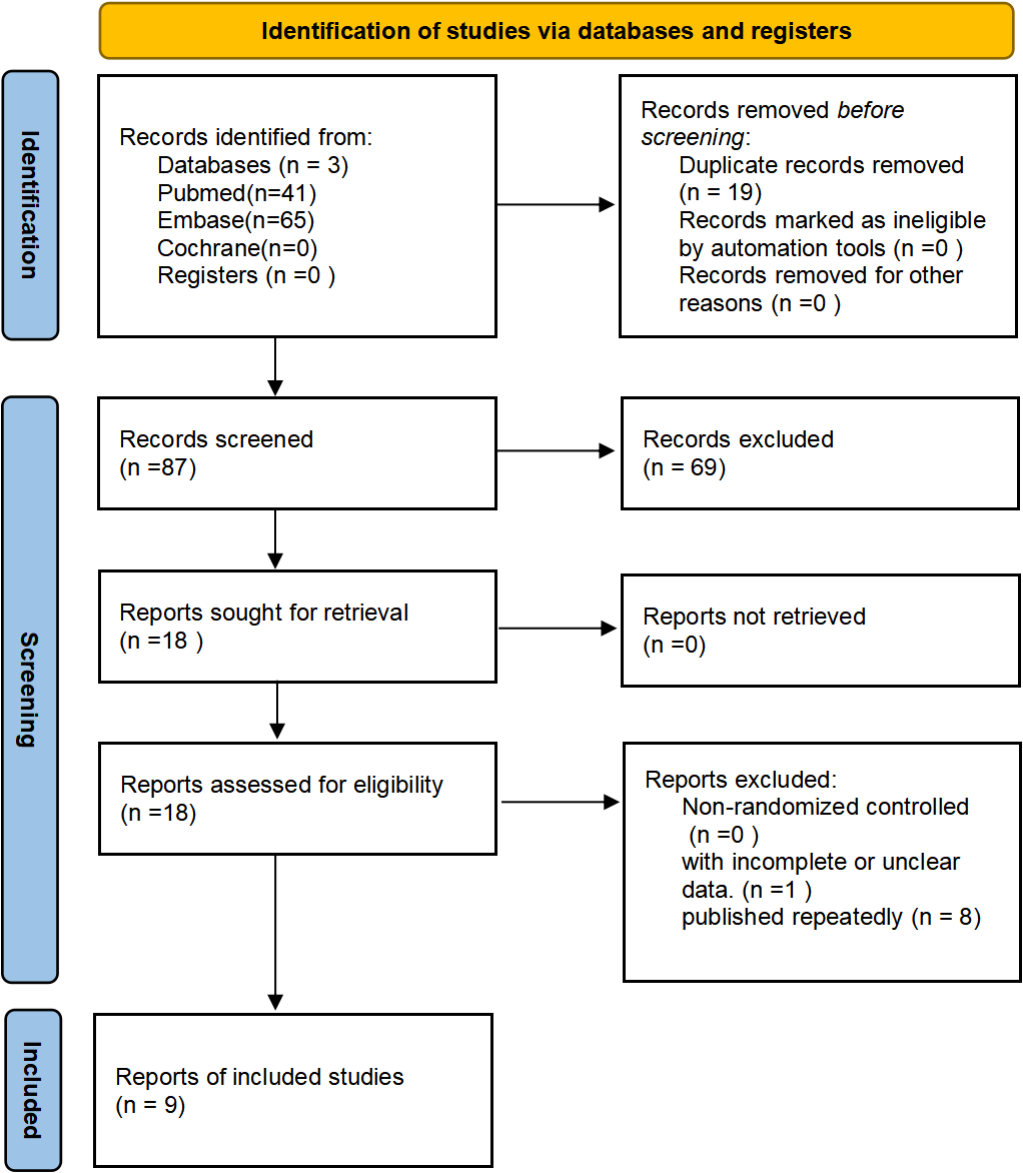
Flowchart of the details of the study.

A total of 9 clinical studies were included (13–21)with 9818 cases. The literature selection process is shown in **Figure 1**. The included studies were published from 2018 to 2022, with treatment duration ranging from 26-72weeks. The treatment groups were all treated with the maintenance dose of TZP (5,10 or 15mg once-weekly), while 5 studies used placebo in control group, 4 with GLP-1RAs and 2 with insulin (2 studies adopted both placebo and GLP-1 RAs). In 3 studies, TZP was used alone, while the other hypoglycemic agents in combination with TZP were applied in 6 studies. The basic characteristics of the included studies are shown in **Table 1**.

**Table 1:** Study-level and participant baseline characteristics of included RCTs.

Study; Clinical Trials;gov registration No.	No. of participants	Study arms	Body weight, kg(mean ± sd)	Age, years (mean ± sd);	Diseases	Background glucose-lowering therapy	Study duration (weeks)
Frias2021 (13) (SURPASS-2) (NCT03987919)	470	TZP 5mg	33.8 ± 6.85	56.3 ± 10.0	Type 2 diabetes	Metformin	40
	469	TZP 10mg	34.3 ± 6.60	57.2 ± 10.5			
	470	TZP 15mg	34.5 ± 7.11	55.9 ± 10.4			
	469	SMG1mg	34.2 ± 7.15	56.9 ± 10.8			
Jastreboff2022 (14) (NCT04184622)	630	TZP 5mg	37.4 ± 6.63	45.6 ± 12.7	Obesity	–	72
	636	TZP 10mg	38.2 ± 7.01	44.7 ± 12.4			
	630	TZP 15mg	38.1 ± 6.69	44.9 ± 12.3			
	643	Placebo	38.2 ± 6.89	44.4 ± 12.5			
Rosenstock2021 (15) (SURPASS-1) (NCT03954834)	121	TZP 5mg	32.2 ± 7.0	54.1 ± 11.9	Type 2 diabetes	–	40
	121	TZP 10mg	32.2 ± 7.6	55.8 ± 10.4			
	121	TZP 15mg	31.5 ± 5.5	52.9 ± 12.3			
	115	Placebo	31.7 ± 6.1	53.6 ± 12.8			
Prato2021 (16) (SURPASS-4) (NCT03730662)	329	TZP 5mg	32.6 ± 6.06	62.9± 8.6	Type 2 diabetes	Mono-therapy with or any combination of metformin, sulfonylurea, or SGLT2 inhibitor	52
	328	TZP 10mg	32.8 ± 5.51	63.7± 8.7			
	338	TZP 15mg	32.5 ± 5.02	63.7± 8.6			
	1000	IG	32.5 ± 5.55	63.8 ± 8.5			
Dahl2022 (17) (SURPASS-5) (NCT04039503)	116	TZP 5mg	33.6 ± 5.9	62 ± 10	Type 2 diabetes	Insulin glargine ± metformin	40
	119	TZP 10mg	33.4 ± 6.2	60± 10			
	120	TZP 15mg	33.4 ± 5.9	61 ± 10			
	120	Placebo	33.2 ± 6.3	60 ± 10			
Heise2022 (18) (NCT03951753)	45	TZP 15mg	31.28 ± 5.01	61.1± 7.1	Type 2 diabetes	Metformin ± another oral hypoglycemic agent	28
	44	SMG1mg	30.82 ± 3.84	63.7 ± 5.9			
	28	Placebo	32.24 ± 3.96	60.4± 7.6			
Frias2018 (19) (NCT03131687)	55	TZP 5mg	32.9 ± 5.7	57·9± 8.2	Type 2 diabetes	± Metformin	26
	51	TZP 10mg	32·6 ± 5.8	56.5 ± 9.9			
	53	TZP 15mg	32.2 ± 6.2	56.0± 7.6			
	54	DLG 1.5mg	32.4 ± 5.4	58.7 ± 7.8			
	51	Placebo	32.4 ± 6.0	56.6± 8.9			
Inagaki2022 (20) (NCT03861052)	159	TZP 5mg	28.6 ± 5.4	56.8 ± 10.1	Type 2 diabetes	–	52
	158	TZP 10mg	28.0 ± 4.1	56.2± 10.3			
	160	TZP 15mg	28.1± 4.4	56.0± 10.7			
	159	DLG 0.75mg	27.8± 3.7	57.5± 10.2			
Ludvik2021 (21) (SURPASS-3) (NCT038882970)	358	TZP 5mg	33.6± 5.9	57.2± 10.1	Type 2 diabetes	Metformin ± SGLT2 inhibitor	52
	360	TZP 10mg	33.4 ± 6.2	57.4 ± 9.7			
	359	TZP 15mg	33.7 ± 6.1	57.5± 10.2			
	360	ID	33.4± 6.1	57.5± 10.1			

TZP, Tirzepatide; SMG, Semaglutide; DLG, dulaglutide; IG, Insulin glargine; ID, insulin degludec.

All studies were RCTs, 6 were double-blind and 3 were open-label. We used the Cochrane risk-of-bias tool and modified Jadad scale to assess the risk of bias, as shown in **Supplementary Figure 1** and **Supplementary Table 1**. The quality of all studies was all acceptable.

### 3.2 Publication bias

From the funnel plots, there was the obvious publication bias in almost all dose groups between TZP and placebo or GLP1-RAs which may have impacts on the stability of the results. But, the assessment may be not accurate enough as the number of studies is less than 10.(**Supplementary Figures 2**, **3**).

### 3.3 Meta-analyses

**Figure 2:**
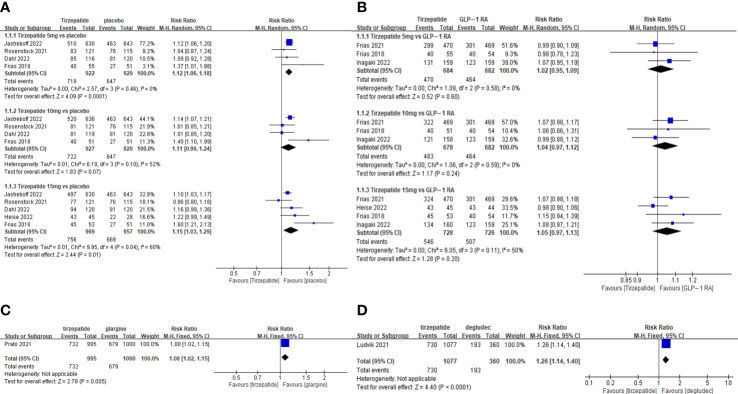
Meta-analysis results for tirzepatide of total adverse drug event: **(A)** tirzepatide vs placebo. **(B)** tirzepatide vs GLP-1RAs. **(C)** tirzepatide vs insulin glargine. **(D)** tirzepatide vs insulin degludec.

**Figure 3:**
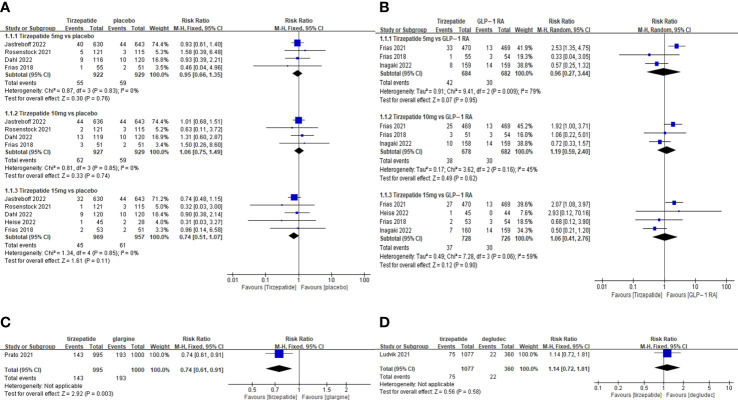
Meta-analysis results for tirzepatide of serious adverse drug event: **(A)** tirzepatide vs placebo. **(B)** tirzepatide vs GLP-1RAs. **(C)** tirzepatide vs insulin glargine. **(D)** tirzepatide vs insulin degludec.

**Figure 4:**
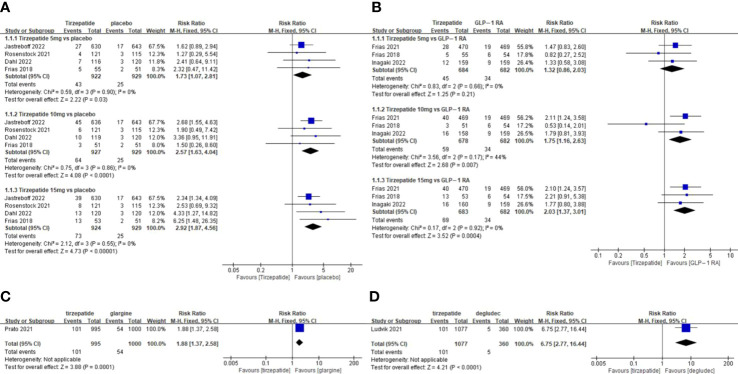
Meta-analysis results for tirzepatide of discontinuation by adverse drug event: **(A)** tirzepatide vs placebo. **(B)** tirzepatide vs GLP-1RAs. **(C)** tirzepatide vs insulin glargine. **(D)** tirzepatide vs insulin degludec.

The TADE incidences of 5mg and 15mg TZP were higher than those of placebo, but with no statistically significant differences in each dose group compared to GLP-1 RAs (**Figure 2**); The odds of SADE were similar between TZP in all groups and GLP-1 RAs (**Figure 3**); The GADE was more frequent with all TZP doses than placebo, but comparable to GLP-1 RAs (**Supplementary Figure 4**). Of which, the incidences of nausea, vomiting and diarrhea were higher than placebo in all dose groups but still consistent with GLP-1 RAs (**Supplementary Figures 5**–**7**); TZP 15mg was associated with more hypoglycemia than GLP-1 RAs (pooled RR=3.83, 95%CI [1.19-12.30], *P=0.02*) (**Supplementary Figure 8**); The odds of injection-site reaction were higher in TZP 5mg and 10mg groups than those of placebo, but all dose groups were the same as GLP-1 RAs (**Supplementary Figure 9**); The risk of discontinuation by ADE was significantly higher in all does groups than placebo. Compared with GLP-1 RAs, more participants receiving TZP 10 mg (pooled RR=1.75, 95%CI [1.16-2.63], *P=0.007*) and 15mg (pooled RR=2.03, 95%CI [1.37-3.01], *P=0.0004*) experienced the discontinuation (**Figure 4**). TZP had lower odds of hypoglycemia compared to glargine (pooled RR=0.40, 95%CI [0.31-0.51], *P<0.00001*) and degludec (pooled RR=0.21, 95%CI [0.11-0.38], *P<0.00001*) (**Supplementary Figure 8**), but similar odds of injection-site reaction with insulin; The TADE, GADE and discontinuation were less usual in the insulin groups than TZP.

### 3.4 Chi-square analysis results of different doses of TZP for adverse drug event

Our study showed that increasing the dose of TZP could not promote the emergence of TADE,SADE,GADE and hypoglycemia (*P>0.05*), suggesting that there may be no dose- dependence; Compared with 5mg, 10mg and 15mg of TZP were also associated with more frequent of nausea (*P<0.001*), discontinuation (*P<0.05*) and injection-site reaction (*P<0.01*), but 10mg and 15mg were equivalent (*P>0.05*).It indicates the obvious dose-dependence in range from 5 to10mg; The incidence of vomiting was 5 mg<10 mg (*P<0.01*), 10 mg<15 mg (*P<0.05)*, 5 mg<15 mg (*P<0.001*), illustrating the significant dose-dependence in range of 5-15 mg; For diarrhea, there were no differences between 5mg and 10mg (*P>0.05*), and the same results were observed between 10mg and 15mg (*P>0.05*). But TZP 15mg can lead to more diarrhea than 5mg (*P<0.05*),which revealed that there may be a weak dose-dependence within 5-15mg (**Table 2**).

**Table 2:** Chi-square analysis results of different doses of Tirzepatide for adverse drug event.

Adverse drug event	Comparator arm	No. of studies	Events/total	Events/total	P	Chi^2^
Total adverse drug event	5mg vs 10mg	8	1599/2238	1654/2242	0.487	0.483
	10mg vs 15mg	8	1654/2242	1736/2296	0.588	0.293
	5mg vs 15mg	9	1599/2238	1736/2296	0.215	1.539
Serious adverse drug event	5mg vs 10mg	8	173/2238	171/2242	0.905	0.014
	10mg vs 15mg	8	171/2242	146/2296	0.118	2.441
	5mg vs 15mg	9	173/2238	146/2296	0.093	2.829
Gastrointestinal adverse drug event	5mg vs 10mg	3	252/646	292/641	0.130	2.292
	10mg vs 15mg	3	292/641	296/644	0.928	0.008
	5mg vs 15mg	3	252/646	296/644	0.108	2.578
Nausea	5mg vs 10mg	9	376/2238	515/2242	<0.001#	17.894
	10mg vs 15mg	9	515/2242	568/2296	0.273	1.2
	5mg vs 15mg	9	376/2238	568/2296	<0.001#	28.443
Diarrhea	5mg vs 10mg	9	344/2238	395/2242	0.086	2.944
	10mg vs 15mg	9	395/2242	423/2296	0.557	0.346
	5mg vs 15mg	9	344/2238	423/2296	0.021*	5.342
Vomiting	5mg vs 10mg	9	145/2238	197/2242	0.007#	7.261
	10mg vs 15mg	9	197/2242	246/2296	0.047*	3.934
	5mg vs 15mg	9	145/2238	246/2296	<0.001#	21.721
Hypoglycemia	5mg vs 10mg	7	64/2183	58/2191	0.578	0.309
	10mg vs 15mg	7	58/2191	74/2243	0.215	1.537
	5mg vs 15mg	8	64/2183	74/2243	0.496	0.465
Injection-site reaction	5mg vs 10mg	8	46/2238	79/2242	0.004#	8.419
	10mg vs 15mg	8	79/2242	96/2296	0.268	1.226
	5mg vs 15mg	9	46/2238	96/2296	<0.001#	15.862
Discontinuation due to adverse drug event	5mg vs 10mg	8	145/2238	185/2242	0.035*	4.450
	10mg vs 15mg	8	185/2242	204/2251	0.375	0.785
	5mg vs 15mg	8	145/2238	204/2251	0.003#	8.942

*P<0.05, ^#^P<0.01.

### 3.5 Sensitivity analysis

Considering the possible influence of the blind on the results, open-label studies were excluded (in TZP vs GLP-1 RAs subgroups). The results are generally stable, except for “injection-site reaction”. The heterogeneity is mainly from Frias (2021), in which the participants were encouraged to change injection sites constantly, whereas the other studies did not mention this method. This may be the source of heterogeneity. Therefore, there is no evidence of the influence by the blind on the results.

In further analyses, the study of Frias (2018) was found to be with high heterogeneity in many subgroup analyses. This may be the initial dose and dose escalation of TZP were different from the others (In this study, initial dose was 5 mg, instead of 2.5 mg, and at the rate of 2 weeks for dose escalation was faster compared to 4 weeks in other studies), which may lead to more injection-site reactions and GADE. Heise (2022) also showed heterogeneity. It may be due to the small sample size which could produce random errors. In addition, the duration of these two trials are shorter which may also lead to heterogeneity.It is because GADE mainly occurs in the first weeks of administration.The short duration may increase the difference between TZP and placebo groups but not GLP1-RAs. Overall, there was high heterogeneity in some outcomes, most of which could be explained by the differences of interventions among studies (clinical heterogeneity). However, due to the lack of sufficient data, it is difficult to evaluate and analyze the degree of heterogeneity caused by these factors.

## 4 Discussion

In recent years, more has been learned about the safety of GLP-1 RAs, but the safety of TZP, the first dual GLP-1/GIP receptor agonist needs to be further researched as its short history of clinical application. It is currently believed that gastrointestinal events, pancreatitis or elevated serum amylase, cardiac arrhythmias, allergies, injection site reactions, hypoglycemia and acute gallbladder disease could occur during the clinical application of TZP (8).In this systematic review, we have summarized and synthesized the up-to-date RCT results of TZP vs placebo, GLP-1 RAs and basal insulin for ADE evaluation.

The result of this research showed the rates of TADE by TZP with different doses were comparable to those of GLP-1 RAs. The total safety was similar between different dose groups by chi-square tests suggesting that TADE rate was not dose-dependence. This result may not be consistent with some of previous studies (22), possibly due to the inclusion of new research results.

For SADE, the rates of TZP at different doses were similar to placebo, GLP-1 RAs. It seems that the risk of SADE is acceptable. However, the definition of SADE may be not accordant among studies, because we noticed that “Covid-19 infection” was included in some studies, but was not involved in others (It may be affected by the timing and region of the pandemic of Covid-19). In addition, the different characteristics of patients may also have the influence. In some studies, the patients were older or with more complications which may lead to more SADE and death themselves. These may reduce the differences the of SADE between TZP and controlled agents. Therefore, the final conclusion needs further confirmation.

GLP-1 acts as an inhibitor of gastric and pancreatic motility and maintains postprandial glucose stability. Thus, GADE is the most common, which may not only be one of the reasons for its effect on weight loss *via* reducing appetite but also for the discomfort felt by patients. The odds of nausea, vomiting, and diarrhea by taspoglutide and lixisenatide are more than 80%, and over 50% by exenatide with the obvious dose-dependence (23). The mechanism is considered as the activating the central nervous system (CNS) GLP-1 receptors most likely located in brain stem (area postrema) (23), and gastrointestinal GLP-1 receptors (24). Encouragingly, this study did not show that TZP had a higher risk of GADE compared to GLP-1 RAs, which was similar to some results of the previous study (10). Theoretically, TZP acts on GLP-1 receptor on one hand, and on the other hand when the agent activates GIP receptor, it has no direct effects on gastrointestinal motility and secretory function which does not increase the rate of GADE (8). The result of this study seems to prove the viewpoint that GIP receptor activation does not cause additional GADE. However, there are still some issues which need to be explored. Previous studies (25–27) showed that reducing refined sugar and fat intake may help to reduce GADE by GLP-1 RAs, but whether these methods are suitable for TZP needs to be confirmed by further research.

The current view is that, consistent with GLP-1 RAs, the higher dose of TZP is associated with more GADE (22). But our result did not seem to support this point. It is notably that the amount of studies included was small (only three) and the final conclusion may not be sufficiently reliable. Although it seem to be contradictory, the derivation may be interesting. Nausea, vomiting and diarrhea were more common in high dose groups although they accounted for the majority of GADE, but were not all the symptoms (13, 15, 19). If there is no dose-dependence of total GADE, it may show the remaining GADE (including abdominal pain, bloating, constipation and decreased appetite, etc) may be with unobvious dose-dependence, which also needs to be further confirmed.

GADE of GLP-1 RAs occurs mainly in the first weeks of treatment and then subsides or stabilizes over time, but severe symptoms can also lead to discontinuation. The discontinuation rate of GLP-1 RAs is currently considered to be 0%-15%, with exenatide slightly lower than semaglutide or dulcolactone (28). An important finding of this study was that discontinuation rates of TZP in all does groups were significantly higher than placebo, with more participants discontinuation at or over 10 mg than GLP-1 RAs, mainly due to intolerable GADE (13, 15–17, 20, 21, 29), which is consistent with the result of the chi-square analysis.The GADE can lead to discontinuation including nausea, diarrhea, vomiting, indigestion, abdominal pain, loss of appetite and constipation.The majority are common in GADE. It may be a disturbing and an alarming signal of TZP safety. Compared to the previous study (10), this study found higher discontinuation of TZP than GLP-1 RAs starting at 10 mg rather than 15 mg, which may affect the suitable dose confirmation of TZP. It might be due to the strengthened effect of GIP on GLP-1 receptors in CNS which can lead to more severe GADE (30). However, it also seems to be difficult to elucidate this mechanism distinctly at this time, as the results of the basal research are not consistent.

Although the overall safety of insulin is better than TZP, however, considering the better efficacy, potential role of cardiovascular protection and convenient administration way, the safety profile may not prevent TZP to replace insulin in T2DM patients. Compared with placebo and GLP-1 RAs, the results of this study showed that TZP did not increase the risk of hypoglycemia. It suggests that TZP, like GLP-1 RAs, might not induce hypoglycemia at appropriate doses. However, more hypoglycemia patients were found in 15 mg TZP group than GLP-1 RAs, and the reason for this needs to be further investigated. Notably, in some studies included, TZP was combined with other anti-diabetic agent usage which may have impacts on the final results. There is still controversy whether TZP itself can cause hypoglycemia. The previous opinion was that TZP alone may not cause hypoglycemia (8). However, some studies believed that the risk still exists (14, 15). Combining with these findings, it suggests that the risk of hypoglycemia by TZP especially at high dose, should not be completely ignored.

This study did not discuss the risk of pancreatitis, tumors, cardiovascular event and hepatobiliary diseases by TZP, as the odds of these ADE were too low to be trusted in clinical trials. These can only be researched by signal mining and retrospective cohort studies in future (31). Meanwhile, due to the insufficient data form the existing trials, some results of this study were affected by small sample sizes thus more confirmation is required. In addition, the higher clinical heterogeneity of some results could lead to instability which also need more well-designed studies.

## 5 Conclusion

The safety profile of TZP was overall acceptable, similar to GLP-1 RAs. However, TZP 15 mg may be associated with more hypoglycemia than GLP-1 RAs. Meanwhile, it should be noted that more discontinuations were discovered by TZP at 10 mg or over than GLP-1 RAs due to GADE. In addition, TADE, SADE, GADE and hypoglycemia were not dose-dependence; but nausea, vomiting, diarrhea, discontinuation and injection-site reaction were dose-dependence among specific dose ranges. The optimal dose of TZP should be determined by balancing the efficacy and safety. Moreover, some outcomes in this study were with high heterogeneity due to the differences in trial design and they may be with biases and need the further confirmation.Thus, more well-designed trials are needed to control the confounding factors and ensure adequate sample size.

## Data availability statement

The original contributions presented in the study are included in the article/**Supplementary Material**. Further inquiries can be directed to the corresponding author.

## Author contributions

Conceptualization YW, ZM, MY. Methodology, YW. Software, HW, CX. Validation SZ, YW, ZM, MY. Formal analysis, YW. Investigation, YW, ZM, MY. Resources, HW, SZ. Data Curation, YW, ZM, MY. Writing – original draft preparation, ZM, MY. Writing – review and editing YW, HW, SZ. Visualization, YW, CX. Supervision, YW. Project administration YW, HW. All authors contributed to the article and approved the submitted version.

## Supplementary material

Supplementary Figure 1 Assessment of the risk of bias in included studies with cochrane domain-based quality assessment tool.

Supplementary Figure 2 Publication bias of safety for tirzepatide vs placebo (Funnel plot). **(A)** total adverse drug event **(B)** serious adverse drug event **(C)** gastrointestinal adverse drug event **(D)** nausea **(E)** vomiting **(F)** diarrhea **(G)** discontinuation by adverse drug event **(H)** hypoglycemia **(I)** injection-site reaction.

Supplementary Figure 3 Publication bias of safety for tirzepatide vs GLP-1RAs (Funnel plot). **(A)** total adverse drug event **(B)** serious adverse drug event **(C)** gastrointestinal adverse drug event **(D)** nausea **(E)** vomiting **(F)** diarrhea **(G)** discontinuation by adverse drug event **(H)** hypoglycemia **(I)** injection-site reaction.

Supplementary Figure 4 Meta-analysis results for tirzepatide of gastrointestinal adverse drug event: **(A)** tirzepatide vs placebo. **(B)** tirzepatide vs GLP-1RAs.

Supplementary Figure 5 Meta-analysis results for tirzepatide of nausea: **(A)** tirzepatide vs placebo. **(B)** tirzepatide vs GLP-1RAs. **(C)** tirzepatide vs insulin Glargine **(D)** tirzepatide vs insulin Degludec.

Supplementary Figure 6 Meta-analysis results for tirzepatide of vomiting: **(A)** tirzepatide vs placebo. **(B)** tirzepatide vs GLP-1RAs. **(C)** tirzepatide vs insulin Glargine **(D)** tirzepatide vs insulin Degludec.

Supplementary Figure 7 Meta-analysis results for tirzepatide of diarrhea: **(A)** tirzepatide vs placebo. **(B)** tirzepatide vs GLP-1RAs. **(C)** tirzepatide vs insulin Glargine **(D)** tirzepatide vs insulin Degludec.

Supplementary Figure 8 Meta-analysis results for tirzepatide of hypoglycemia: **(A)** tirzepatide vs placebo. **(B)** tirzepatide vs GLP-1RAs. **(C)** tirzepatide vs insulin Glargine **(D)** tirzepatide vs insulin Degludec.

Supplementary Figure 9 Meta-analysis results for tirzepatide of injection-site reaction: **(A)** tirzepatide vs placebo. **(B)** tirzepatide vs GLP-1RAs. **(C)** tirzepatide vs insulin Glargine **(D)** tirzepatide vs insulin Degludec.
