# Supplementary figures and images for: A systematic review of the safety of tirzepatide-a new dual GLP1 and GIP agonist - is its safety profile acceptable?
Source: Front Endocrinol (Lausanne). 2023 Mar 27;14:1121387. doi: 10.3389/fendo.2023.1121387 (PMC10084319; doi:10.3389/fendo.2023.1121387)

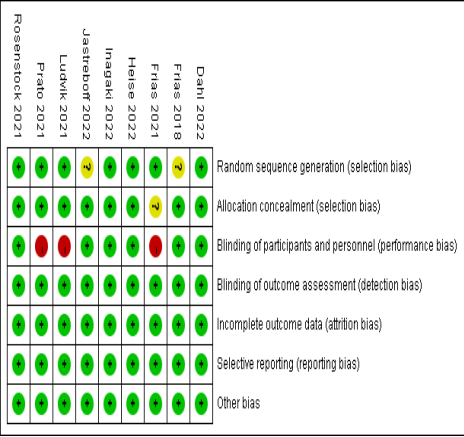

Supplement: Supplementary Figure 1 — Assessment of the risk of bias in included studies with cochrane domain-based quality assessment tool. [file Image_1.tif]

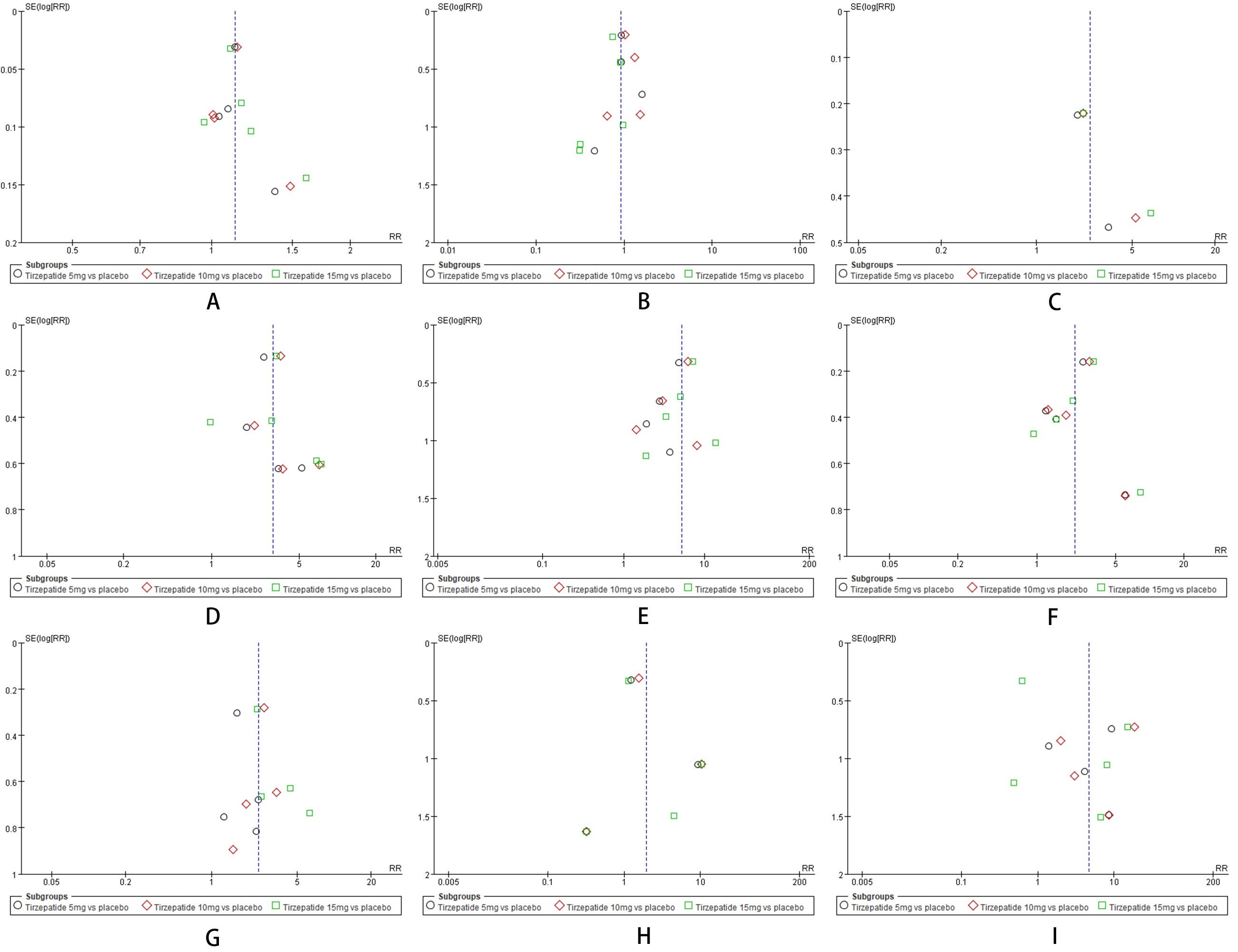

Supplement: Supplementary Figure 2 — Publication bias of safety for tirzepatide vs placebo (Funnel plot). (A) total adverse drug event (B) serious adverse drug event (C) gastrointestinal adverse drug event (D) nausea (E) vomiting (F) diarrhea (G) discontinuation by adverse drug event (H) hypoglycemia (I) injection-site reaction. [file Image_2.tif]

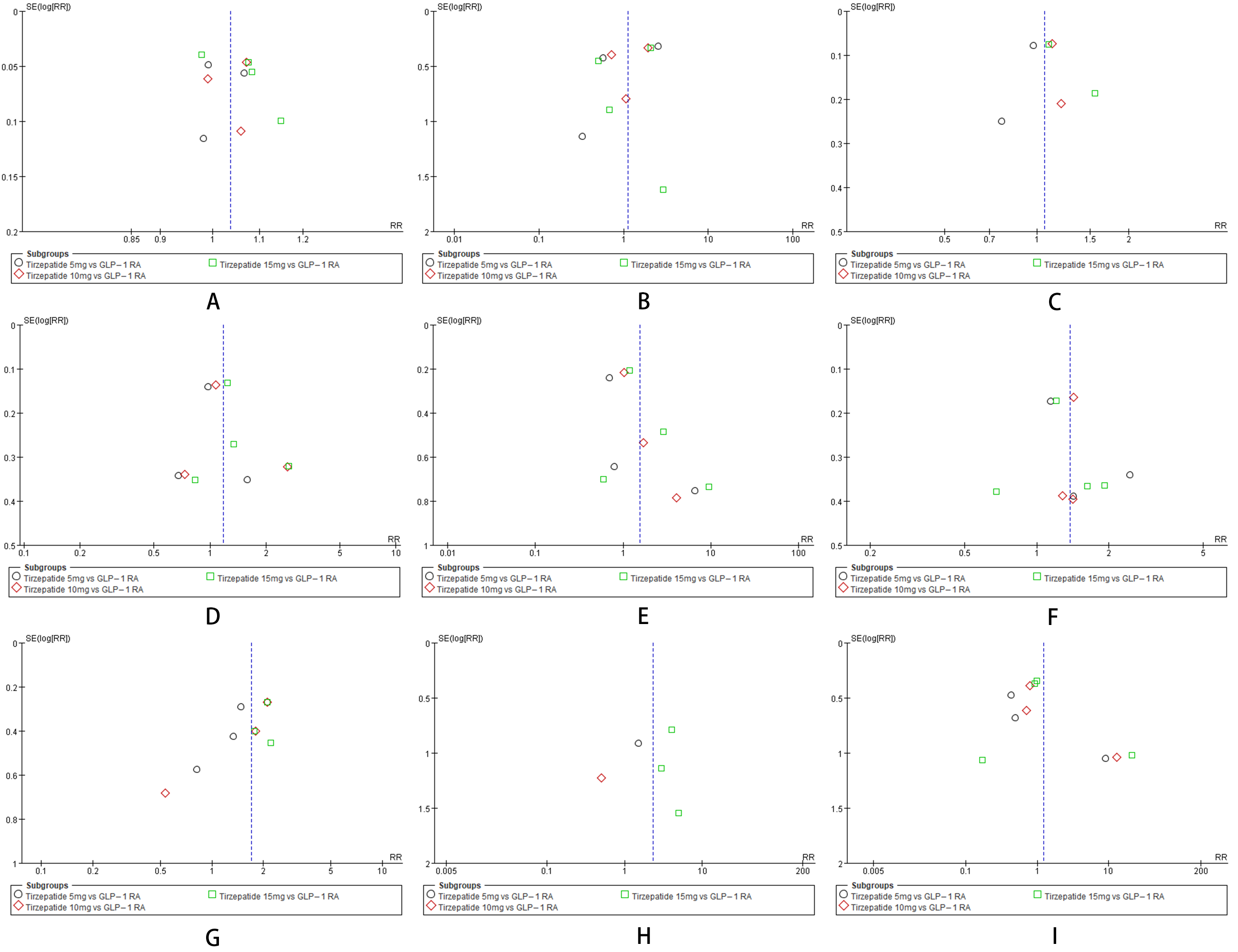

Supplement: Supplementary Figure 3 — Publication bias of safety for tirzepatide vs GLP-1RAs (Funnel plot). (A) total adverse drug event (B) serious adverse drug event (C) gastrointestinal adverse drug event (D) nausea (E) vomiting (F) diarrhea (G) discontinuation by adverse drug event (H) hypoglycemia (I) injection-site reaction. [file Image_3.tif]

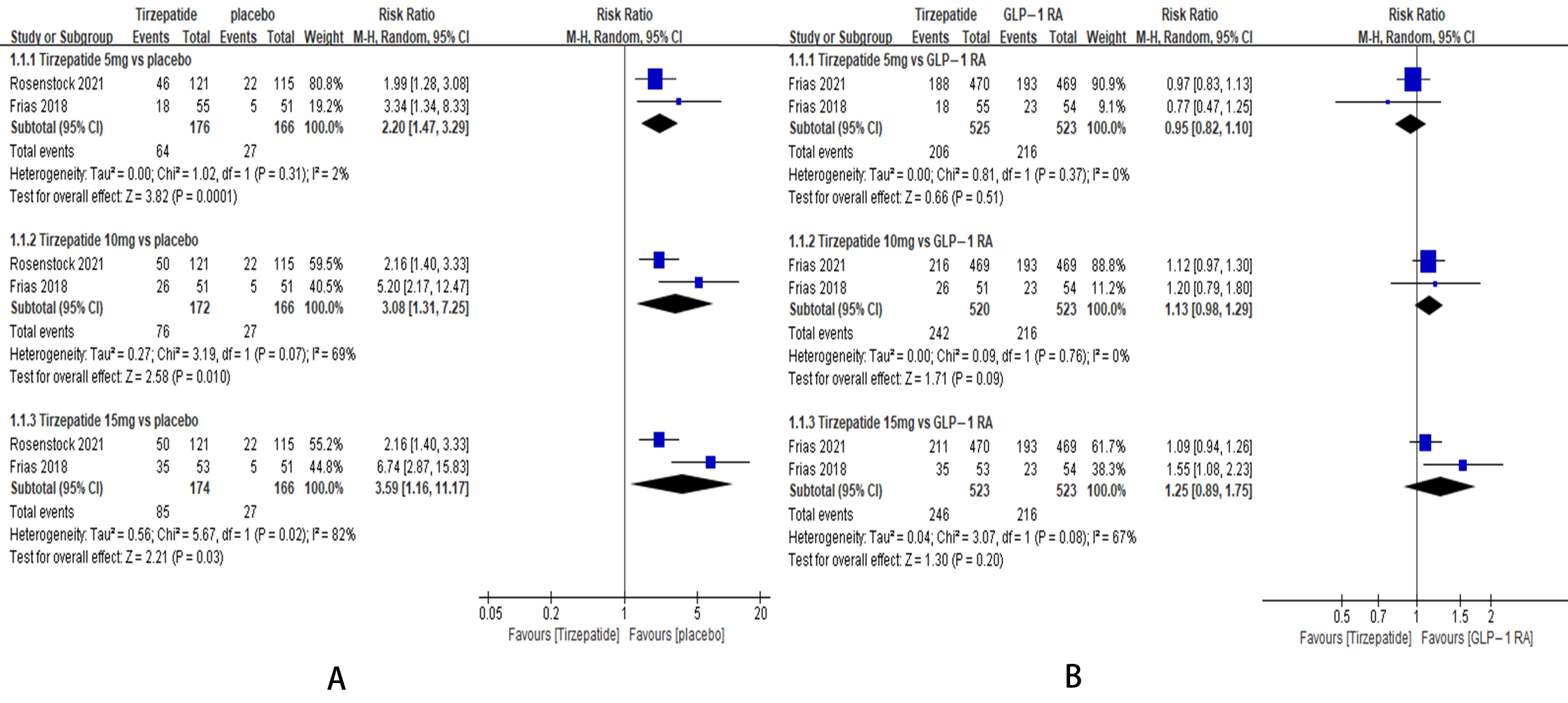

Supplement: Supplementary Figure 4 — Meta-analysis results for tirzepatide of gastrointestinal adverse drug event: (A) tirzepatide vs placebo. (B) tirzepatide vs GLP-1RAs. [file Image_4.tif]

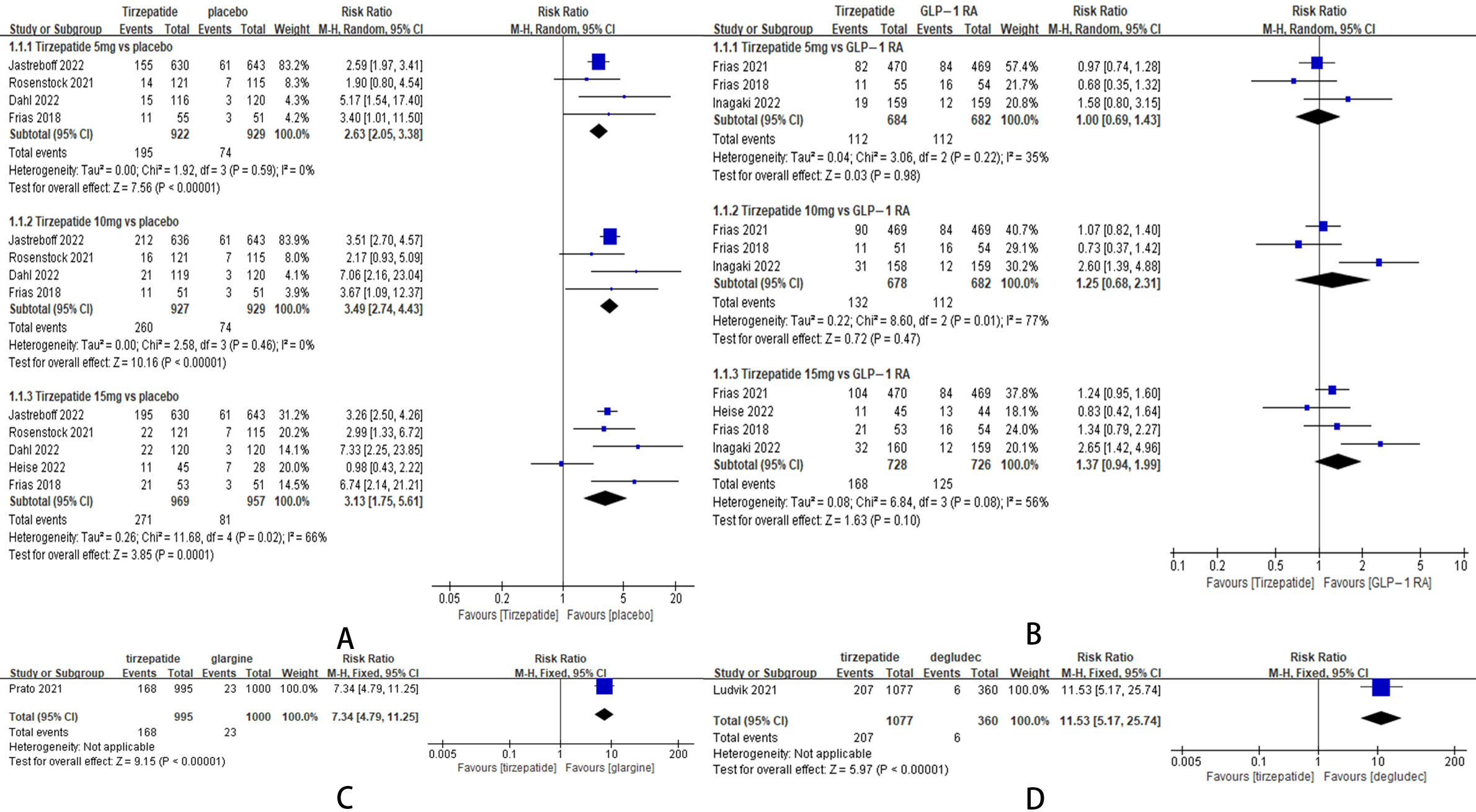

Supplement: Supplementary Figure 5 — Meta-analysis results for tirzepatide of nausea: (A) tirzepatide vs placebo. (B) tirzepatide vs GLP-1RAs. (C) tirzepatide vs insulin Glargine (D) tirzepatide vs insulin Degludec. [file Image_5.tif]

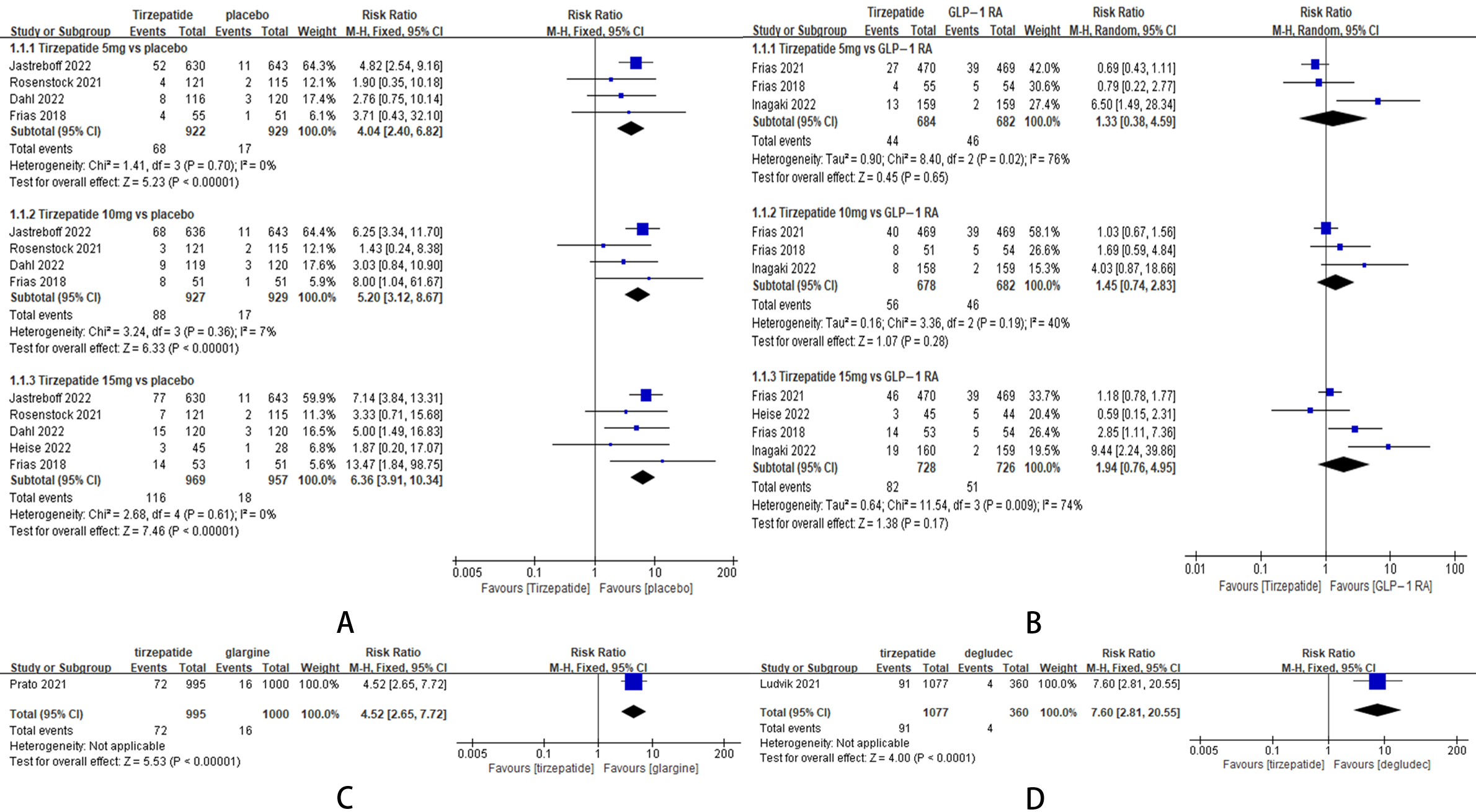

Supplement: Supplementary Figure 6 — Meta-analysis results for tirzepatide of vomiting: (A) tirzepatide vs placebo. (B) tirzepatide vs GLP-1RAs. (C) tirzepatide vs insulin Glargine (D) tirzepatide vs insulin Degludec. [file Image_6.tif]

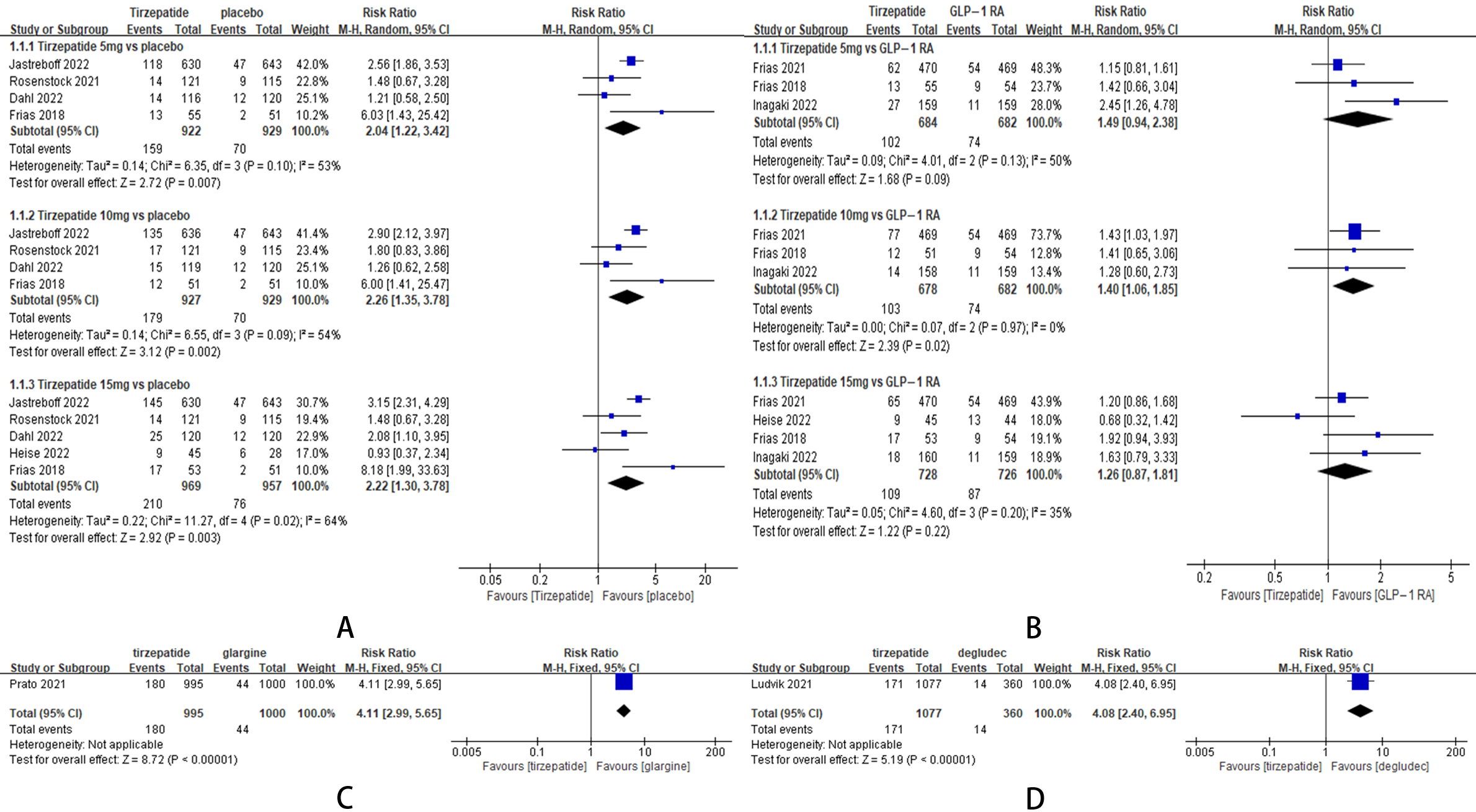

Supplement: Supplementary Figure 7 — Meta-analysis results for tirzepatide of diarrhea: (A) tirzepatide vs placebo. (B) tirzepatide vs GLP-1RAs. (C) tirzepatide vs insulin Glargine (D) tirzepatide vs insulin Degludec. [file Image_7.tif]

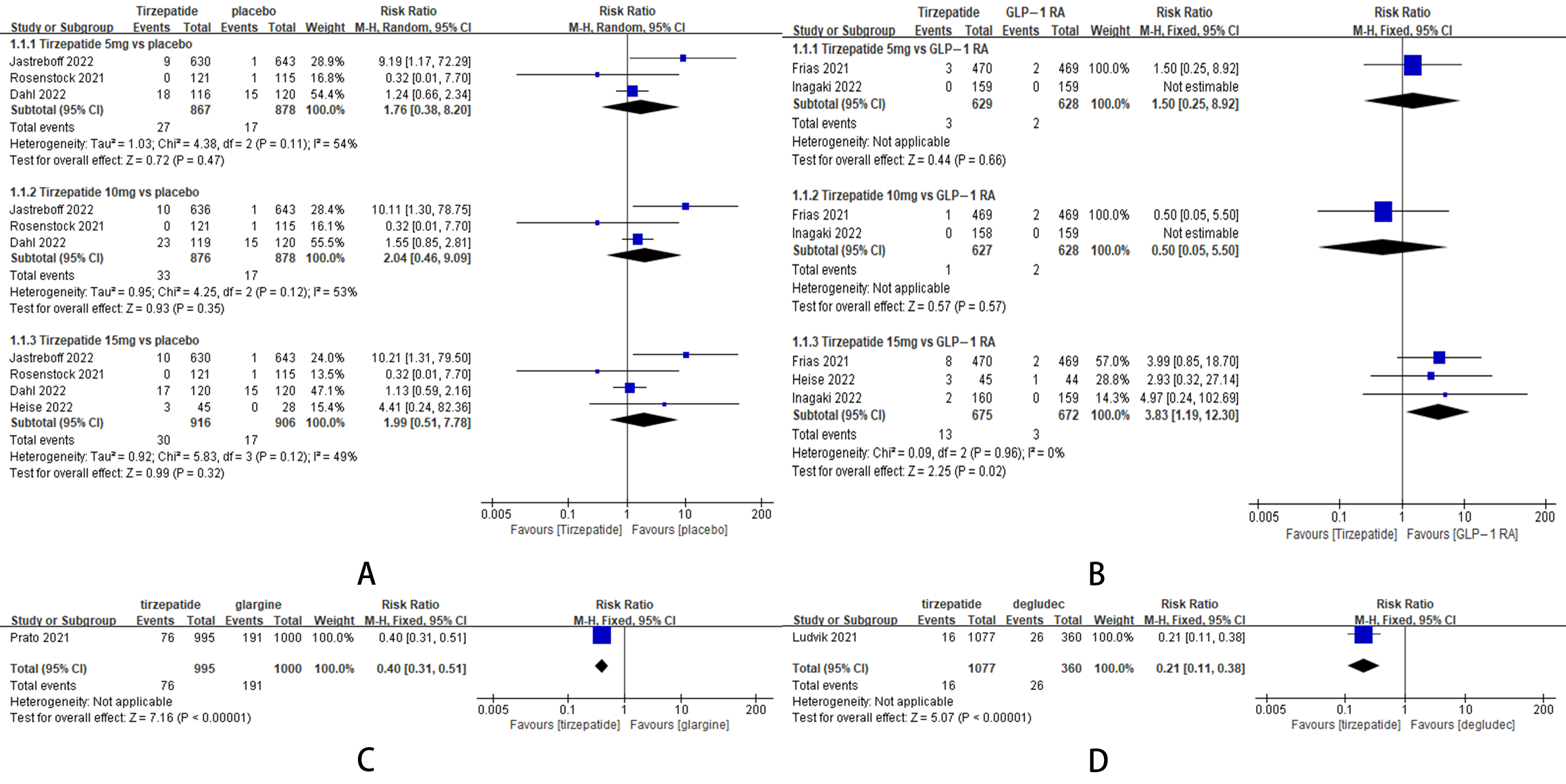

Supplement: Supplementary Figure 8 — Meta-analysis results for tirzepatide of hypoglycemia: (A) tirzepatide vs placebo. (B) tirzepatide vs GLP-1RAs. (C) tirzepatide vs insulin Glargine (D) tirzepatide vs insulin Degludec. [file Image_8.tif]

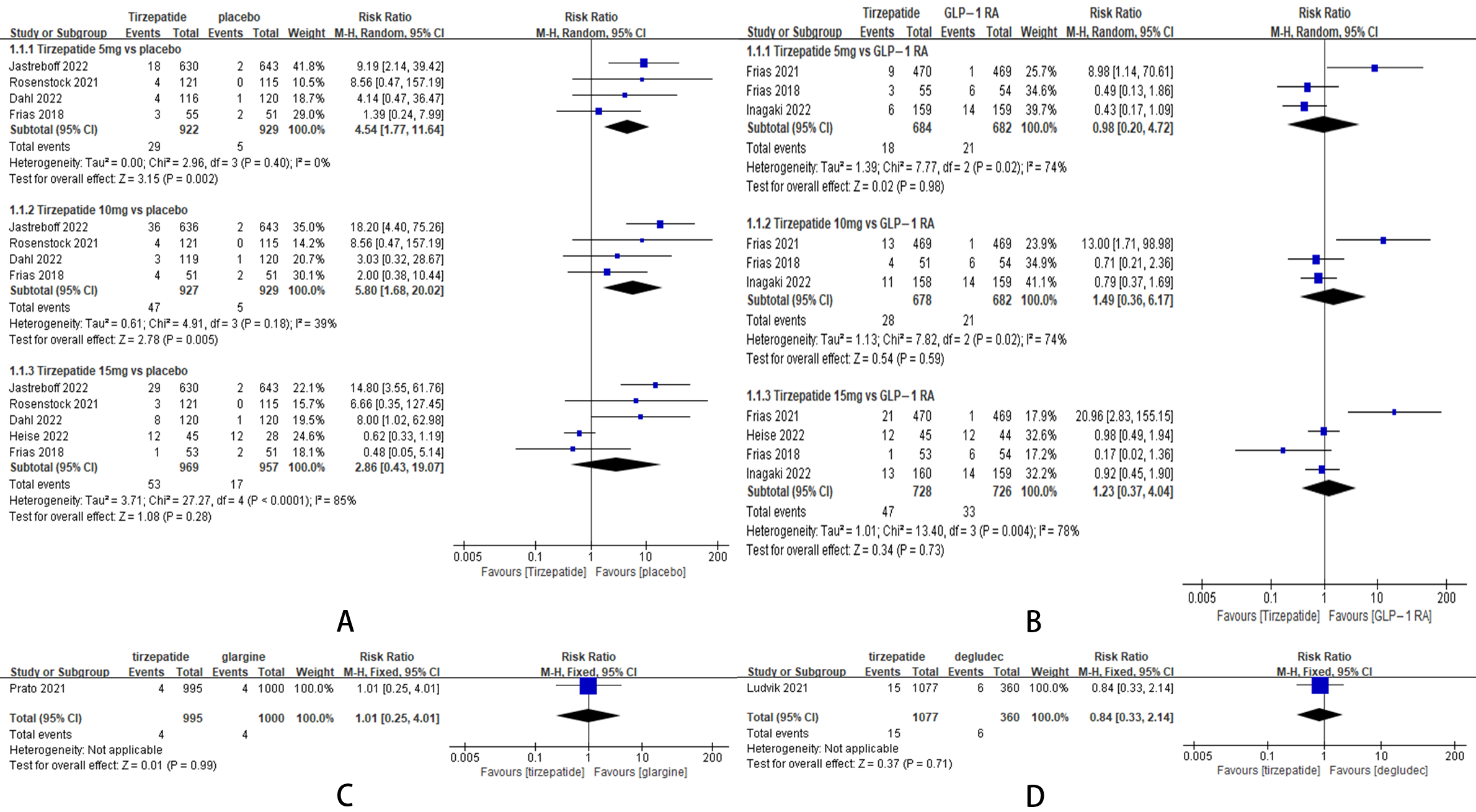

Supplement: Supplementary Figure 9 — Meta-analysis results for tirzepatide of injection-site reaction: (A) tirzepatide vs placebo. (B) tirzepatide vs GLP-1RAs. (C) tirzepatide vs insulin Glargine (D) tirzepatide vs insulin Degludec. [file Image_9.tif]
