# Supplementary material for: A systematic review of the safety of tirzepatide-a new dual GLP1 and GIP agonist - is its safety profile acceptable?
Source: Front Endocrinol (Lausanne). 2023 Mar 27;14:1121387. doi: 10.3389/fendo.2023.1121387 (PMC10084319; doi:10.3389/fendo.2023.1121387)
Supplement: Supplementary file 10 [file Table_1.doc]

| Study | Randomisation | Allocation concealment | Double-blind | Dropouts and withdrawals | Jadad score |
| --- | --- | --- | --- | --- | --- |
| Frias2021 | Yes | Yes | Open-label | Yes | 4 |
| Jastreboff2022 | Yes | Yes | Yes | Yes | 6 |
| Rosenstock2021 | Yes | Yes | Yes | Yes | 7 |
| Prato2021 | Yes | Yes | Open-label | Yes | 5 |
| Dahl2022 | Yes | Yes | Yes | Yes | 7 |
| Heise2022 | Yes | Yes | Yes | Yes | 7 |
| Frias2018 | Yes | Yes | Yes | Yes | 6 |
| Inagaki2022 | Yes | Yes | Yes | Yes | 7 |
| Ludvik2021 | Yes | Yes | Open-label | Yes | 5 |

Supplement table 1 Assessment of the risk of bias
